# Consequences of elevated temperature and *p*CO_2_ on insect folivory at the ecosystem level: perspectives from the fossil record

**DOI:** 10.1002/ece3.2203

**Published:** 2016-05-30

**Authors:** Ellen D. Currano, Rachel Laker, Andrew G. Flynn, Kari K. Fogt, Hillary Stradtman, Scott L. Wing

**Affiliations:** ^1^ Departments of Botany and Geology & GeophysicsUniversity of WyomingLaramieWyoming; ^2^ Department of Geology and Environmental Earth ScienceMiami UniversityOxfordOhio; ^3^ Department of GeologyBaylor UniversityWacoTexas; ^4^ Department of PaleobiologySmithsonian InstitutionWashingtonDistrict of Columbia

**Keywords:** Bighorn Basin, herbivory, legumes, Paleogene, Paleocene–Eocene Thermal Maximum, plant‐insect interactions

## Abstract

Paleoecological studies document the net effects of atmospheric and climate change in a natural laboratory over timescales not accessible to laboratory or ecological studies. Insect feeding damage is visible on well‐preserved fossil leaves, and changes in leaf damage through time can be compared to environmental changes. We measured percent leaf area damaged on four fossil leaf assemblages from the Bighorn Basin, Wyoming, that range in age from 56.1 to 52.65 million years (Ma). We also include similar published data from three US sites 49.4 to ~45 Ma in our analyses. Regional climate was subtropical or warmer throughout this period, and the second oldest assemblage (56 Ma) was deposited during the Paleocene–Eocene Thermal Maximum (PETM), a geologically abrupt global warming event caused by massive release of carbon into the atmosphere. Total and leaf‐chewing damage are highest during the PETM, whether considering percent area damaged on the bulk flora, the average of individual host plants, or a single plant host that occurs at multiple sites. Another fossil assemblage in our study, the 52.65 Ma Fifteenmile Creek paleoflora, also lived during a period of globally high temperature and *p*CO_2_, but does not have elevated herbivory. Comparison of these two sites, as well as regression analyses conducted on the entire dataset, demonstrates that, over long timescales, temperature and *p*CO_2_ are uncorrelated with total insect consumption at the ecosystem level. Rather, the most important factor affecting herbivory is the relative abundance of plants with nitrogen‐fixing symbionts. Legumes dominate the PETM site; their prevalence would have decreased nitrogen limitation across the ecosystem, buffering generalist herbivore populations against decreased leaf nutritional quality that commonly occurs at high *p*CO_2_. We hypothesize that nitrogen concentration regulates the opposing effects of elevated temperature and CO_2_ on insect abundance and thereby total insect consumption, which has important implications for agricultural practices in today's world of steadily increasing *p*CO_2_.

## Introduction

**Figure 1:**
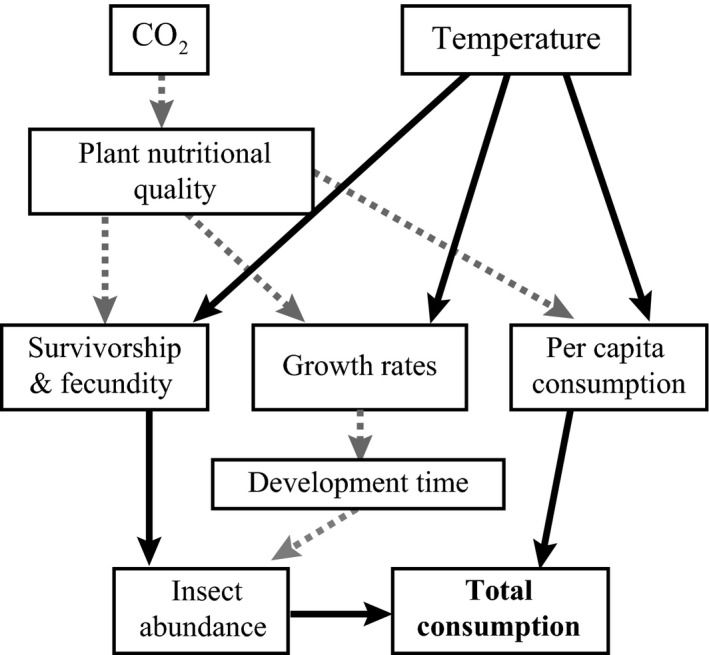
Predicted effects of elevated *p*CO_2_ and temperature on insect herbivores. Solid black arrows indicate positive correlations and gray dashed arrows indicate negative correlations. Decreased plant nutritional quality at high *p*CO_2_ is due to foliar increases in C:N ratio, tannins, and toughness.

Fossil fuel combustion and land use change since the start of the industrial revolution have drastically altered the composition of the atmosphere, increasing carbon dioxide concentration from ~280 ppm in 1750 to ~400 ppm in 2015 (http://www.esrl.noaa.gov/gmd/ccgg/trends/). Global temperature rose 0.85°C between 1880 and 2012, and further addition of CO_2_ is expected to yield a total warming of 2–3°C by the end of the 21st century (Stocker et al. 2013). These changes will have major effects on terrestrial ecosystems and two groups that dominate them, plants and insect herbivores. Ecological and physiological observations and experiments have documented the current state of plant–insect food webs and provide insight about their response to elevated CO_2_ and temperature (see review by DeLucia et al. 2012; Fig. 1). Because these studies are limited to short timescales and small areas, though, paleoecological results are essential for understanding how these processes will play out over larger areas and longer periods of time. Here, we discuss the response of plant–insect herbivore interactions to intervals of elevated *p*CO_2_ and temperature in the geologic past.

Temperature directly affects insect population dynamics and geographic distributions. As ectotherms, insects are highly sensitive to ambient temperatures and may respond very quickly to its fluctuations (Robinet and Roques 2010). Warming accelerates insect metabolism and growth rate, thereby decreasing development time and mortality rates (Bale et al. 2002). This, in turn, may increase the number of generations per year and population density, as has already been documented for many different insects groups and geographic locations (e.g., Hansen et al. 2001; Harrington et al. 2001; Gomi et al. 2007; Jonsson et al. 2009). Temperature change also removes or relocates barriers to insect range expansion; for example, nonmigratory European butterfly species have shifted their ranges northward, at times extending beyond the range of their original, primary host plants (Hill et al. 1999; Parmesan et al. 1999). During the last decade in North America, outbreaks of mountain pine beetle, gypsy moth, spruce beetle, and spruce budworm have progressed northwards, likely in response to milder winters (Logan et al. 2003). A potential negative impact of warming on insect fitness is the disruption of host–insect phenological synchrony (reviewed in Robinet and Roques 2010).

A series of meta‐analyses have been conducted to find generalized responses of extant plants and insect herbivores to elevated *p*CO_2_ (Bezemer and Jones 1998; Zvereva and Kozlov 2006; Stiling and Cornelissen 2007; Robinson et al. 2012). In general, plants show an increase in carbohydrates and a decline in protein levels under elevated *p*CO_2_, resulting in a 19% average increase in C:N ratio (Robinson et al. 2012). Tannin concentration and leaf toughness also increase with elevated *p*CO_2_ (Stiling and Cornelissen 2007; Robinson et al. 2012), and collectively, these factors decrease the nutritional quality of leaves. These changes should increase herbivore consumption (compensatory feeding) and reduce herbivore fitness. Studies by Stiling and Cornelissen (2007) and Robinson et al. (2012) document increased relative consumption rates (16.5% and 14% increases, respectively) and total consumption (9.2% and 16.7%), but the two disagree on the response of insect abundance to elevated *p*CO_2_. Stiling and Cornelissen (2007) report a 22% decrease, whereas Robinson et al. (2012) conclude that abundance changes are highly variable among guilds and feeding strategies. There is no evidence of carbon dioxide directly affecting insects.

Meta‐analyses are dominated by controlled feeding experiments, which generally involve a single insect species being fed a single plant species, and cannot address multitrophic interactions, high variability among species or genotypes, and feeding or oviposition preferences of insects. Free‐air concentration enrichment (FACE) experiments avoid some of these problems, and, interestingly, some of the most extensive FACE experiments have conflicting results. Hamilton et al. (2004) and Knepp et al. (2005) observed a decrease in community‐level herbivory at elevated *p*CO_2_ (~550 ppm in Hamilton et al. 2004; and ~577–586 ppm in Knepp et al. 2005) in the deciduous understory of a forest dominated by loblolly pine, sweet gum, and yellow poplar. In contrast, Couture et al. (2015) documented an 88% increase in canopy damage rates, equating to a ~167% loss in annual primary productivity, at elevated *p*CO_2_ (~560 ppm) in aspen and birch stands. Given the varied results of the FACE experiments, it is likely that the response of plant–insect associations to elevated *p*CO_2_ depends strongly on interactions among environmental variables. Zvereva and Kozlov (2006) suggest that increasing temperature ameliorates the negative impact of elevated *p*CO_2_ on insect fitness, and Leuzinger et al. (2011) hypothesize that response magnitude decreases with higher‐order interactions, longer time periods, and larger spatial scales. However, recent experiments manipulating *p*CO_2_, temperature, and drought in a heathland ecosystem showed that including three drivers accentuates, rather than attenuates, biotic responses (Scherber et al. 2013).

The scarcity of studies that manipulate multiple climate variables in natural systems highlights the utility of paleoecological studies in understanding biotic response to future climate changes. Paleoecological studies consider the end result of climate change on whole ecosystems (host plants, herbivores, predators, parasites, symbionts) and therefore do not have the same limitations as experiments or short‐term ecological studies. Paleoecological studies are, however, limited by the nature of the geologic record. For studies of pre‐Quaternary ecosystems, intervals between samples are commonly 10^4^–10^5^ years, and fossil plant deposits are generally dominated by the more robust, woody components of the ecosystem. Well‐preserved leaf fossils can be effectively differentiated into unique morphospecies, but the lack of attached reproductive structures makes it difficult to assign these to taxonomic groups. Last, the magnitude of *p*CO_2_ changes in the fossil record remains difficult to interpret.

The purpose of this study was to examine how elevated temperature and *p*CO_2_ affected insect herbivory in ancient forest ecosystems. We measured the percent of leaf area damaged in a temporal sequence of four fossil floras that span an interval of high global temperature and *p*CO_2_, ~56–52 million years ago. These fossil sites have already been used to document significant positive correlations between temperature and the number of insect damage types observed and the frequency of damaged leaves (Currano et al. 2010). We also compared our measurements with similar data from the literature. Here, we focus on leaf area damaged, the metric most commonly used in ecological and experimental studies. This allows more direct integration of paleoecological results with the extensive literature on modern plant–insect responses to elevated temperature and carbon dioxide.

## Materials and Methods

### Paleocene–Eocene climate

**Figure 2:**
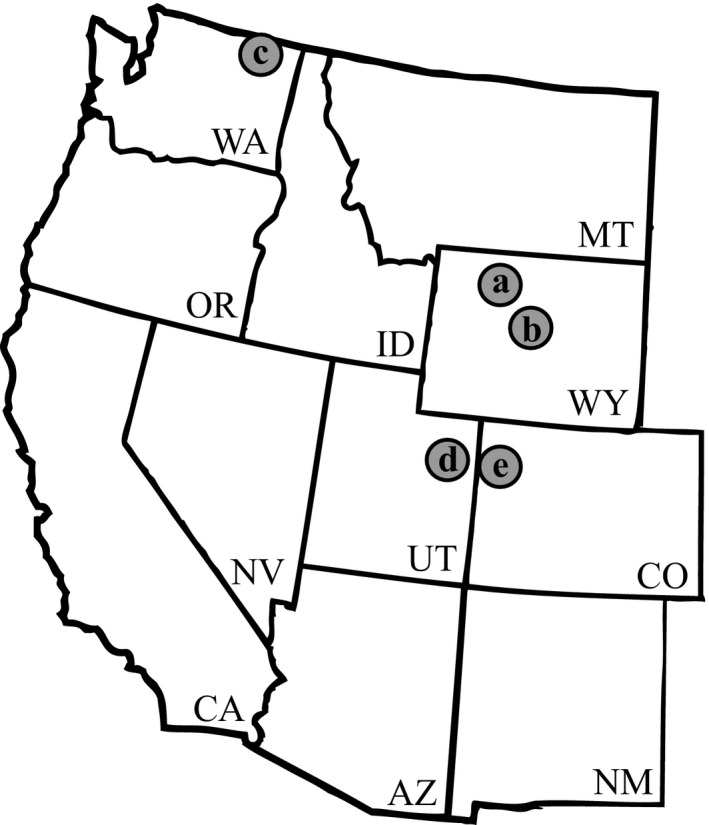
Late Paleocene and early Eocene fossil sites at which leaf area damaged has been measured, including the Fifteenmile Creek (Early Eocene Climatic Optimum [EECO]), PN, Hubble Bubble (Paleocene–Eocene Thermal Maximum), and Daiye Spa sites in the Bighorn Basin (a; this study), an EECO site in the Wind River Basin (b; this study), the 49.4 Ma Republic site with an estimated mean annual temperature (MAT) of 13°C (c; Labandeira 2002, Royer et al. 2007), the 47.3 Ma Bonanza flora whose MAT is estimated to be 14.3°C (d; Wilf et al. 2001; Royer et al. 2007), and the 43 to 47 Ma Parachute Creek flora that grew at an estimated MAT of 19.5°C (e; Smith 2008).

Leaf compression fossils were collected from four sites in the Bighorn Basin of northwestern Wyoming and range in age from 56.1 to 52.65 Ma (late Paleocene to early Eocene Epochs; Table 1; Fig. 2). Paleotemperature trends during these epochs have been documented both globally, using deep‐sea oxygen isotope measurements (e.g., Zachos et al. 2001, 2008), and in the Bighorn Basin, using paleobotanical and isotopic analyses (Wing et al. 2000; Currano et al. 2010). Beginning in the late Paleocene, temperatures gradually rose to their highest levels of the last 65 Ma, a sustained warm interval called the Early Eocene Climatic Optimum (EECO; 51–53 Ma). Also captured in our study sites is the Paleocene–Eocene Thermal Maximum (PETM) (reviewed by McInerney and Wing 2011 and Wing and Currano 2013), a geologically abrupt warming event caused by the injection of thousands of gigatons of carbon into the atmosphere and ocean (Zeebe et al. 2009). Global temperature increased by 4°–8°C, and the combined temperature and *p*CO_2_ changes considerably affected biotic systems (e.g., Thomas and Shackleton 1996; Gingerich 2003; Gibbs et al. 2006; Currano et al. 2008; Smith et al. 2009; Secord et al. 2012; Wing and Currano 2013). Temperature and *p*CO_2_ returned to background levels about 200 thousand years after the onset of the PETM (Murphy et al. 2010).

**Table 1:** Bighorn Basin sampling summary. PN and Daiye Spa were chosen to represent background temperature and *p*CO_2_ conditions during our study interval and therefore are not associated with a climate event. Mean annual temperature (MAT) was determined using paleobotanical analyses, as described in the references. The specimens measured column gives the number of fossil leaves for which leaf area damaged was measured

Flora	USNM locality number	Epoch, climate event	Age (Ma)	MAT (°C)	Lithology	No. plant species at 450 leaves	Specimens measured
Fifteenmile Creek	42400–42406	Eocene, Early Eocene Climatic Optimum	52.65	22.2 ± 2^a^	Laterally extensive carbonaceous shale	17.6 ± 4.9^d^	130
PN	37560	Eocene	53.4	15.8 ± 2.2^a^	Mud/silt lens	12.1 ± 3.7^d^	148
Hubble Bubble	42384	Eocene, Paleocene–Eocene Thermal Maximum	56	20.1 ± 2.8^b^	Mud/silt lens	20.0 ± 5.0^d^	253
Daiye Spa	41643	Paleocene	56.1	16.4 ± 2.9^c^	Mud/silt lens	14.7 ± 4.0^d^	265

a Wing et al. (2000).

b Wing et al. (2006).

c Currano et al. (2008).

d Currano et al. (2010).

While temperature changes are well constrained across our study interval, *p*CO_2_ is more challenging to interpret. A variety of *p*CO_2_ proxies have been developed, and there is general consensus on large‐scale trends over the last 65 million years (Beerling and Royer 2011). However, proxies disagree on the magnitude of past atmospheric carbon dioxide concentrations. Estimates for the early Eocene range from approximately twice present‐day levels (Smith et al. 2010; Hyland and Sheldon 2013) to over 2700 ppm (Yapp 2004). Similarly, the presence of a carbon isotope excursion during the PETM provides clear evidence of carbon forcing, but uncertainty in the carbon source and discrepancies in the geologic record make it difficult to constrain *p*CO_2_ or rates of carbon release. Models based on geologic data from Spitsbergen conclude that the injection of carbon into the atmosphere occurred over 10–20 thousand years, and *p*CO_2_ increased from ~800 ppm to ~1500 ppm for a methane source or to ~4200 ppm for an organic carbon source (Cui et al. 2011). Rates of carbon release are estimated at 0.3 and 1.7 Pg/year, respectively (Cui et al. 2011). A similarly designed study using core data from the Bighorn Basin concluded that there were two distinct pulses of carbon release, each lasting <2000 years and with an average release rate of 0.9 Pg/year (Bowen et al. 2015). Despite the uncertainties in determining precise *p*CO_2_ levels in the geologic past, temperature and *p*CO_2_ are coupled in natural systems over long time intervals, and intervals of higher temperature likely coincide with higher *p*CO_2_. Our study sites Hubble Bubble and Fifteenmile Creek capture the PETM and EECO, respectively, providing contrasting rates of change in *p*CO_2_ and temperature.

### Study sites and fossil collections

All four sites are in the fluvially deposited Willwood Fm., and each can be characterized by estimated age and local depositional environment (Table 1). We determined site ages by measuring their stratigraphic elevation and interpolating between levels of known age assuming uniform sediment accumulation rates between dated levels. The three oldest floral assemblages were collected from lenticular mud/silt units, interpreted as pond deposits that formed in abandoned channels. The youngest assemblage was excavated from thin, silty claystone intervals within a laterally extensive carbonaceous shale deposit and is interpreted as distal overbank deposits onto a wet floodplain during intervals of high sediment discharge. Differences in fossil leaf preservation across sites should be minimal given the similarity in rock type.

Fossil leaves were excavated using standard bench quarrying techniques. During fossil excavation, all identifiable leaf specimens were scored for the presence/absence of distinct insect damage morphotypes, and these results were published in Currano et al. (2010). Leaf specimens were divided into informal but unique morphotypes using shape and venation characters (Ellis et al. 2009), and whenever possible were assigned to formally named taxa. A representative subset of fossils was collected at each site and is curated in the Department of Paleobiology, National Museum of Natural History, Smithsonian Institution, which also holds the locality data. We used this subset for leaf area damaged analyses.

### Leaf area damaged analyses

**Figure 3:**
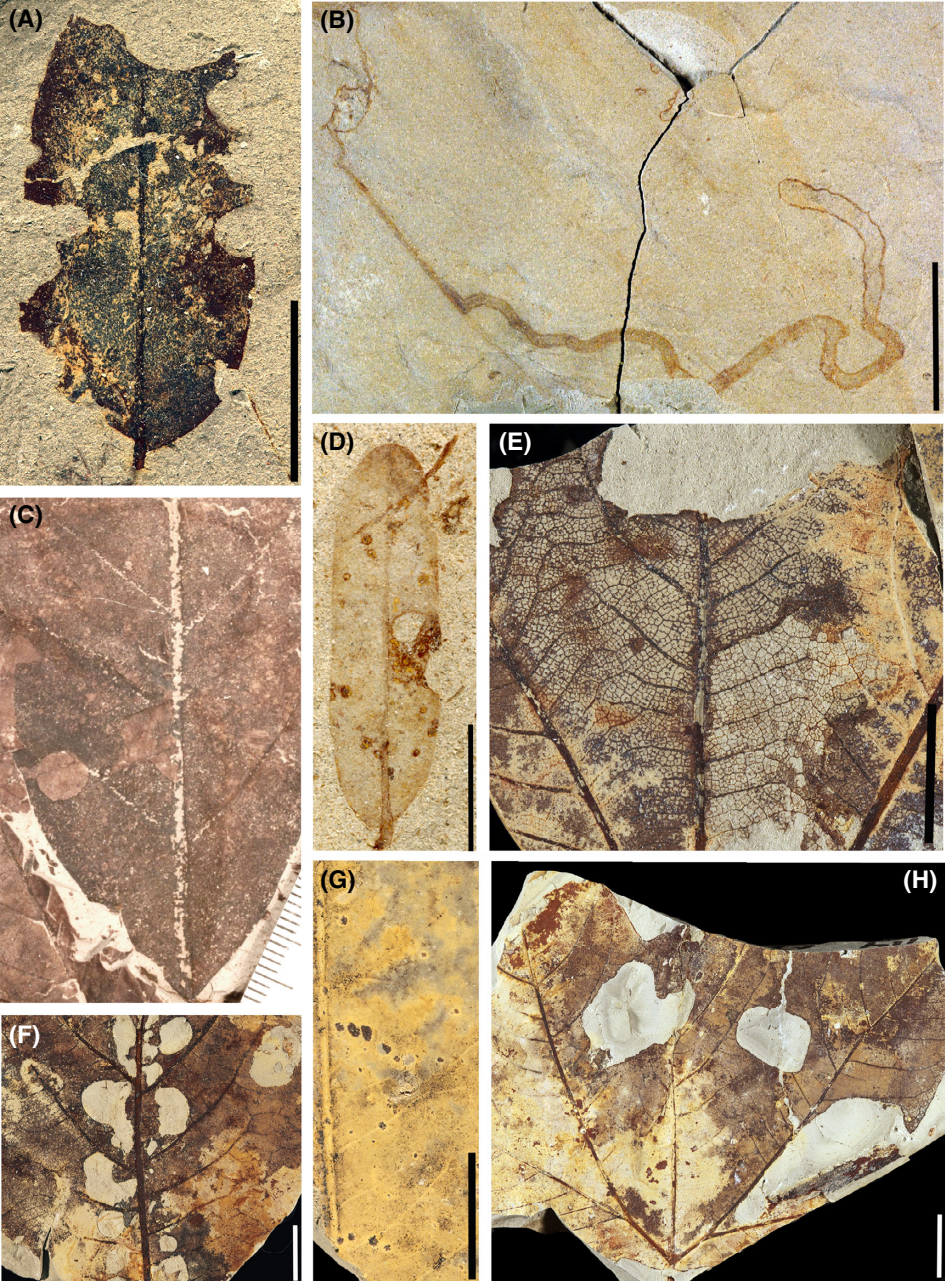
Representative fossil leaves with insect herbivore damage. (A) Fabaceae sp. WW040 (USNM 618001B), collected from the PN site, with margin feeding damage (DT12 and DT13). (B) Dicot sp. WW005 (USNM 618007), collected from Hubble Bubble, with serpentine leaf mine DT40. (C) Lauraceae sp. WW061 (USNM 618002) collected from Fifteenmile Creek, which shows surface feeding DT29. (D) Fabaceae sp. WW001 (USNM 618006) with hole feeding (DT2), margin feeding (DT12), and galls (DT80). (E), *Macginitiea gracilis* (USNM 618003A), collected from the PN site, has skeletonization (DT16), hole feeding (DT3), and margin feeding (DT13). (F) *Macginitiea gracilis* (USNM 618004A), collected from PN, shows a unique form of hole feeding (DT57) in addition to skeletonization (DT16). (G) *Copaifera* sp. (USNM 618008B), also collected from PN, has several galls (DT80). (H), *Macginitiea gracilis* (USNM 618005) from PN, shows large‐scale hole feeding (DT5). Scale bars in A, B, E, F, G, and H are 1 cm, and scale bar in D is 0.5 mm. The ticks on the ruler in C are millimeters.

Insect folivory, including external foliage feeding (e.g., hole feeding, margin feeding, skeletonization, and surface feeding), mines, and galls, is recognizable on well‐preserved leaf compression fossils (Fig. 3). Areas of insect chewing damage are distinguished from postabscission damage by the presence of a reaction rim delineating the damaged area. This thickened, upraised structure, generally composed of parenchymatous callus, is produced as an insect feeds on a living plant and is preserved in the fossil record (Wilf and Labandeira 1999).

All specimens collected as part of the Currano et al. (2010) study were photographed with a metric scale. We measured the area of each damage type and the total surface area of each leaf using the computer program ImageJ (http://imagej.nih.gov/ij/). To do this, a specific number of pixels were set to represent a known length (generally 1 cm). With the scale set, accurate measurements could be made of both the surface area of the leaves and the area of damage. Damage along the margin of the leaf (margin feeding) was measured by drawing a straight line along the missing margin of the leaf, to minimize extrapolation of leaf area. Fossil leaves with excessive insect damage, such as a missing lobe or apex, were excluded. Although this leads to an underestimation of percent leaf area damaged, the shape and size of leaf tissue missing cannot be consistently extrapolated in these circumstances. Less than three leaves from each site were deemed “excessively damaged,” and so this exclusion should not affect our results. Adding the area of all the damage types within each leaf yielded a total area damaged for each leaf. Percent damage for each site was calculated by dividing the total area of damaged leaf tissue for all species by the total leaf area measured for all species. Percent damage for individual species at each site was calculated in a parallel manner for species with more than ten measured specimens.

Although the fossil collections that were used in the leaf area damaged analyses should be similarly biased because the same researcher excavated them, decisions of which specimens to collect are subjective, and excavations were conducted over the course of two field seasons. Therefore, we constructed a dataset in which the frequency of damaged leaves was standardized across sites using subsampling routines in R version 2.15.2 (R Development Core Team; https://www.r-project.org). We selected 5000 subsamples that contained all undamaged leaves measured from a site plus enough damaged leaves from that site to obtain the percent of damaged leaves observed in the unbiased quantitative censuses of Currano et al. (2010; 56% at Fifteenmile Creek, 52% at PN, 56% at Hubble Bubble, and 38% at Daiye Spa). Percent leaf area damaged was calculated for each subsample and these values were averaged for the 5000 subsamples to obtain a standardized leaf area damaged for the site. The list of damaged leaves used in each resample was determined randomly and without replacement. We refer to this as the frequency‐standardized dataset.

Plant species composition varies considerably among sites, and it is therefore necessary to establish whether significant structural differences among leaves at these sites may be driving trends in herbivory. Leaf mass per area (*M*_A_) can be estimated in fossils from petiole width and leaf area using an extensive modern calibration set that demonstrates a robust scaling relationship between petiole width squared and leaf mass, normalized for leaf area (Royer et al. 2007). Species with high *M*_A_ generally have thicker, tougher leaves that are less palatable to insect herbivores, whereas plants with low *M*_A_ tend to have short leaf lifespans and high nutrient concentrations, making them more palatable (Coley and Barone 1996; Royer et al. 2007).

Last, to provide additional context for our results, we searched the literature for fossil data from the late Paleocene through early Eocene of the Western Interior US (Table 2). Analyses of leaf area damaged have been conducted on the 49.4 Ma Republic paleoflora from Washington (Labandeira 2002; Royer et al. 2007), the 47.3 Ma Bonanza paleoflora from Utah (Wilf et al. 2001; Royer et al. 2007), and the Parachute Creek paleoflora of Colorado, whose age is constrained to between 43 and 47 Ma (Smith 2008). We also include new data for *Populus cinnamomoides* from an EECO site in the Wind River Basin, Wyoming, in order to compare damage on a single plant host across time and space. The data for *Populus* come from 28 specimens collected from eight sites (DMNH localities 5097–5104) within the same stratigraphic level and were measured using the protocol described above.

**Table 2:** Published Early Paleogene paleofloras that can be compared to the Bighorn Basin data

Flora	Geographic location and formation	Age (Ma)	Mean annual temperature (°C)	Depositional environment	Specimens measured	Herbivory reference
Parachute Creek	Piceance Creek Basin, SE CO, Green River Fm.	43–47	19.5 ± 3.5	Lacustrine	584^a^	Smith (2008)
Bonanza	Uinta Basin, NE UT, Green River Fm.	47.3	14.3 ± 2.9	Lacustrine	582	Wilf et al. (2001), Royer et al. (2007)
Republic	NE Washington state, Klondike Mountain Fm.	49.4	~13	Lacustrine	749	Labandeira (2002), Royer et al. (2007)

a Includes all leaf fragments >1 cm^2^.

## Results

**Figure 4:**
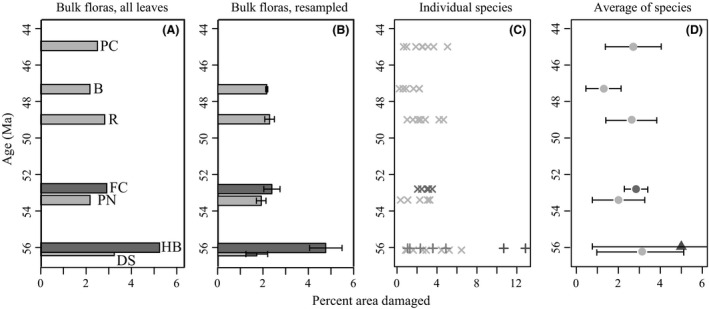
Percent leaf area consumed by insect herbivores through time (Ma, millions of years ago). Age is plotted on the *y*‐axis, as in geologic studies, to show that older sites are stratigraphically below younger sites. (A) Percent leaf area damaged on the four bulk floras measured in this study as well as three previously published fossil floras from the western US (Royer et al. 2007; Smith 2008). All leaves, both damaged and undamaged, were included in this analysis. Site names are abbreviated as, DS: Daiye Spa, HB: Hubble Bubble, PN is the full site name, FC: Fifteenmile Creek, R: Republic, B: Bonanza, PC: Parachute Creek. Site locations are plotted in Figure 2 and summary information given in Tables 1 and 2. Hubble Bubble and Fifteenmile Creek are colored darker gray to emphasize that they are the warmest sites. (B) Percent leaf area damaged on the bulk floras determined by creating 5000 subsamples from each site which have the same percentage of leaves damaged as observed in the quantitative leaf censuses of Currano et al. (2010) and Royer et al. (2007). Bars represent the average of the 5000 subsamples, and error bars are one standard deviation. (C) Percent leaf area damaged on all species with at least 10 leaves on which herbivory could be measured. For clarity, Hubble Bubble (Paleocene–Eocene Thermal Maximum) species are dark gray plus signs. (D) Average percent leaf area damaged for all host species with at least 10 leaves measured at each site. Error bars represent one standard deviation. The Hubble Bubble species average is the dark gray triangle.

**Figure 5:**
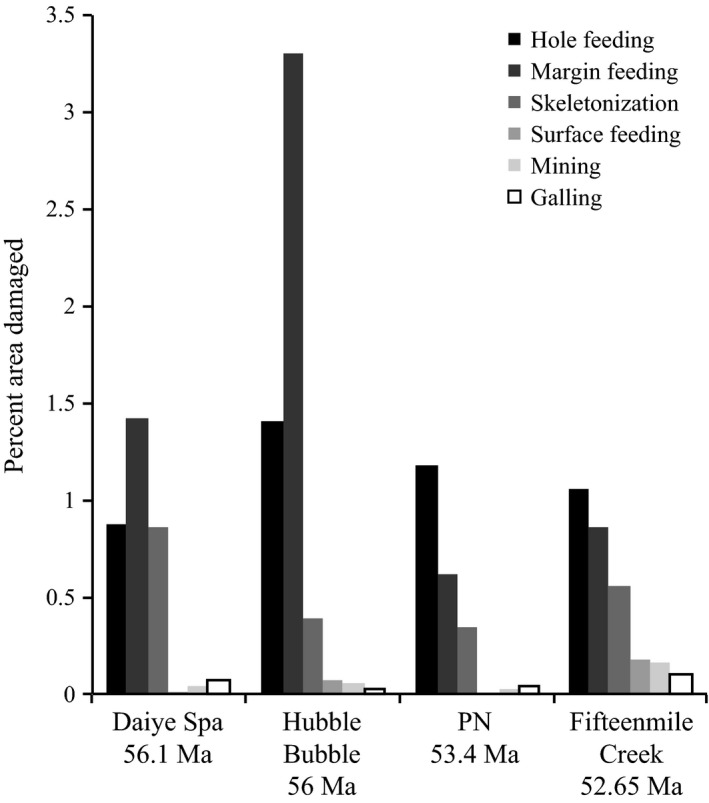
Percent leaf area damaged on the Bighorn Basin bulk floras, partitioned by insect functional feeding group or subgroup. Hole feeding, margin feeding, skeletonization, and surface feeding are subgroups of the external foliage feeding functional feeding group.

The highest percent of damaged leaf area was found at Hubble Bubble, the PETM fossil assemblage (Fig. 4A,B). When all measured leaves are considered, Hubble Bubble (56 Ma) has 5.2% leaf area damaged, compared with the next highest of 3.2% at Daiye Spa (56.1 Ma). The frequency‐standardized dataset, likely a more accurate representation of damage on the fossil floras, shows an even greater difference between Hubble Bubble and all other sites, with 4.79% area damaged on the PETM paleoflora vs. 2.4% at the next highest site (Fifteenmile Creek, the EECO site, 52.65 Ma). The increase in damage at Hubble Bubble is driven by leaf‐chewing insects, particularly margin feeders (Fig. 5; Table 3). The proportion of total damage made by external foliage feeders is highest at Hubble Bubble, and percent leaf area damaged by margin feeding on this paleoflora is double that of any other assemblage. In contrast, damage made by specialist herbivores, particularly leaf miners and gallers, reaches a maximum at Fifteenmile Creek, both in terms of percent of leaf area damaged and proportion of total damage. In particular, the proportion of damage that is mining is about four times higher at Fifteenmile Creek than at Daiye Spa, which is the next highest. Of the four Bighorn Basin sites, Hubble Bubble has the lowest value for galling.

**Table 3:** Proportion of leaf area damaged belonging to each functional feeding group

Flora	Age of flora	External foliage feeding^a^	Mining	Galling
Fifteenmile Creek (Early Eocene Climatic Optimum)	52.65 Ma	0.909	0.058	0.033
PN	53.4 Ma	0.978	0.009	0.016
Hubble Bubble (Paleocene–Eocene Thermal Maximum)	56 Ma	0.984	0.010	0.006
Daiye Spa	56.1 Ma	0.962	0.014	0.023
Adams et al. (2009) temperate forests	Modern	0.970	0.019	0.012
Adams et al. (2009) tropical forests	Modern	0.943	0.040	0.013

a External foliage feeding includes hole feeding, margin feeding, skeletonization, and surface feeding.

Percent area damaged on individual plant hosts is most variable at Hubble Bubble, with values ranging from 1.1% to 12.9% (Fig. 4C). Plant hosts at the other sites range in area damaged from 0.2% to 6.5%. Average area damaged on individual hosts is 5.0% at Hubble Bubble, versus 1.3–3.1% at the other sites. The two most damaged hosts in the Hubble Bubble paleoflora, which are largely responsible for the elevated individual host average (Fig. 4D), are a taxonomically unassigned species (WW005) and a Hernandiaceous species that may belong to the genus *Gyrocarpus* (WW015). The most damaged species at any of the other six sites is a legume species from Daiye Spa (Fabaceae sp. FU750), and the most damaged species at Fifteenmile Creek is *Alnus*. Living relatives of both have nitrogen‐fixing symbionts.

A great deal of floral change occurs throughout the study interval, and no single plant species can be traced from 56.1 to ~45 Ma (Daiye Spa to Parachute Creek). However, the presence and relative abundance of *P. cinnamomoides* (Berry) MacGinitie (Salicaceae) at both Hubble Bubble and Fifteenmile Creek in the Bighorn Basin and an EECO site in the neighboring Wind River Basin permit an interesting comparison of herbivory during these two warm intervals (Table 4). Insect damage census data (Currano et al. 2010; unpublished data) show that the number of distinct damage types observed on a standardized number of leaves is over twice as high at Hubble Bubble than at either EECO site. A major factor driving this is likely the high frequency of damaged leaves at Hubble Bubble, as the number of distinct damage types observed when standardized by damage occurrences is only slightly higher at Hubble Bubble. Leaf area damaged is 1.7× higher on Hubble Bubble *P. cinnamomoides* than on ones from Fifteenmile Creek, both when considering all measured leaves and also the museum collections normalized by percent of leaves damaged observed in the quantitative census data. Damage on the Wind River EECO sample is even lower than that at Fifteenmile Creek.

**Table 4:** Summary of herbivore damage on *Populus cinnamomoides* from all available sites

	Quantitative insect damage censuses					Museum collections: All leaves		Museum collections: Normalized by % damage
	Rank abundance	% of leaves	# DTs at 20 leaves	# DTs at 50 damage occurrences	% of leaves damaged	# leaves	% leaf area damaged	% leaf area damaged
Hubble Bubble (BHB, PETM)	6	4.02	15.1 ± 2.2	16.1 ± 1.5	70 ± 7.2	17	3.57	2.498
Fifteenmile Creek (BHB, EECO)	4	6.97	7.8 ± 1.9	14.9 ± 1.5	43 ± 4.4	24	2.07	1.492
WRB EECO site	4	6.21	6.3 ± 1.5	11.7 ± 0.5	38 ± 4.6	28	1.09	1.037

BHB, Bighorn Basin; WRB, Wind River Basin; EECO, Early Eocene Climatic Optimum; PETM, Paleocene–Eocene Thermal Maximum.

**Figure 6:**
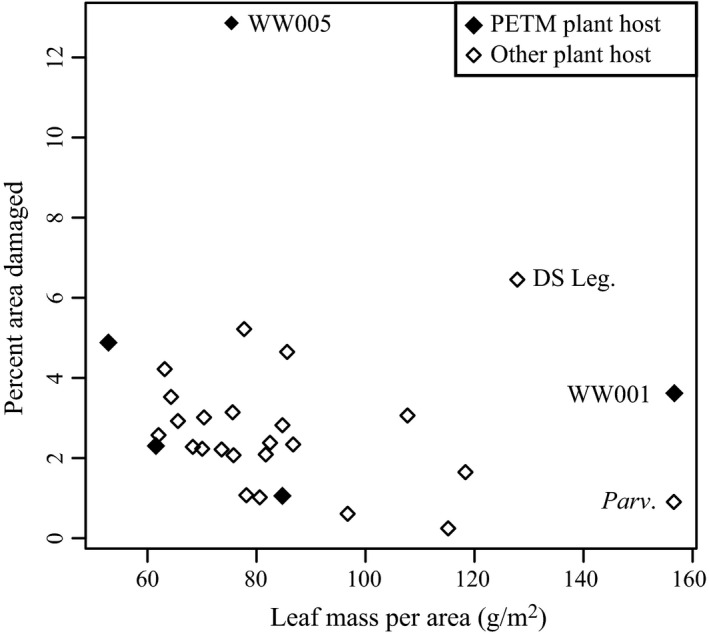
Percent leaf area consumed by insect herbivores vs. leaf mass per area, estimated using the petiole width method of Royer et al. (2007). Data are from our four Bighorn Basin sites (Currano et al. 2010), Republic, and Bonanza (Royer et al. 2007). Each point represents a plant host at a site, and hosts were included only if there were at least two fossils for which leaf mass per area could be estimated and ten fossils that could be measured for leaf area damaged. Outliers are labeled as follows: WW005 is Dicot sp. WW005, DS Leg. is the legume morphotype from Daiye Spa (Fabaceae sp. FU750), WW001 is Fabaceae sp. WW001, and *Parv*. is *Parvileguminophyllum coloradensis* from Bonanza.

Leaf mass per area analyses were used to investigate whether major structural differences among the species present at each site drove fluctuations in herbivory. Figure 6 shows leaf mass per area versus percent area damaged for all species–site pairs with at least ten specimens in the leaf damage analyses and two individuals whose *M*_A_ could be reconstructed. The range in *M*_A_ for the Hubble Bubble species encompasses the combined range of all other sites, and there are no significant among‐site differences in *M*_A_ (an analysis of variance [ANOVA] of *M*_A_ by sites yielded an *F* value of 1.044 and *P *=* *0.41). The Hubble Bubble species with the highest percent area damaged, Dicot sp. WW005, has an estimated M_A_ of 75.4 g/m^2^, placing it in the middle of the Hubble Bubble *M*_A_ range and comparable to many species from other sites. In fact, it is at the high end of the range in *M*_*A*_ at Fifteenmile Creek (65–76 g/m^2^, considering only the species in Fig. 4C). The two species with the highest *M*_A_ are legumes with morphologically similar lepto‐ to nanophyllous leaflets, and the Hubble Bubble species (Fabaceae sp. WW001) has four times more herbivory than the other species (*Parvileguminophyllum coloradensis*), which is from Bonanza.

## Discussion

### Patterns and drivers of Early Paleogene insect herbivory

Total and leaf‐chewing damage are higher at Hubble Bubble, which occurs in the middle of the PETM, than at any other site, whether considering the percent of leaf area damaged across whole floras, the average percent leaf area damaged on individual plant species, or only *Populus cinnamamoides*. To evaluate the direct impact of climate on folivory, though, it is first necessary to assess possible influences of vegetation change through the study interval. Forests in the Bighorn Basin underwent a radical change in composition during the PETM, with thermophilic and probably dry‐tolerant species replacing more mesophytic ones (Wing and Currano 2013). After the PETM, vegetation largely returned to its pre‐PETM composition, although a few immigrant species remained. Despite the drastic species turnover, the consistency in leaf mass per area at all sites suggests no significant differences in leaf toughness existed that would have made PETM species more palatable to folivores than species found at other sites.

**Figure 7:**
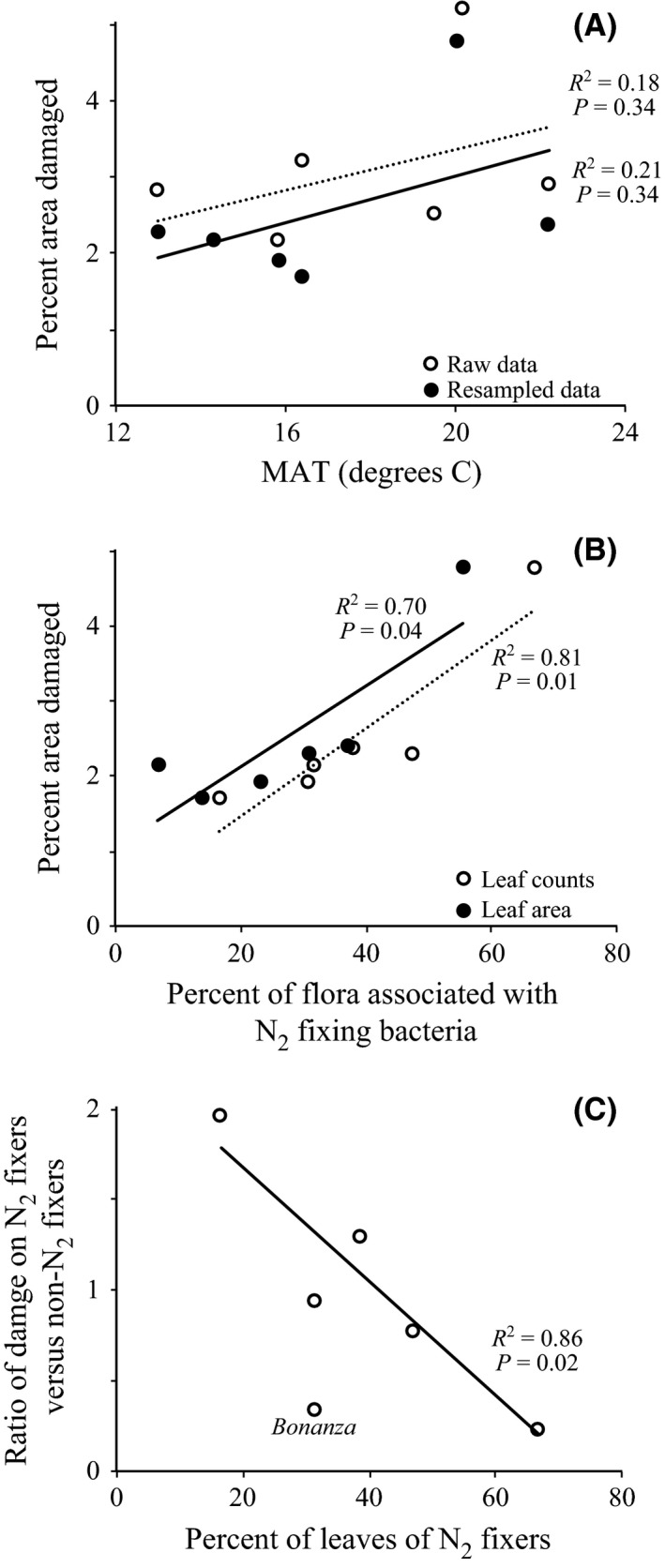
Influence of temperature (A) and plants with N_2_‐fixing bacterial symbionts (B, C) on insect herbivory at the ecosystem level. In (A) and (B), dashed trendlines are for the data indicated by open circles, and solid trendlines are for the data indicated by filled circles. The linear regression given in panel (C) does not include the Bonanza flora. If Bonanza were included, *R* ^2^ drops to 0.51 and is no longer significant (*P* = 0.11).

While it is tempting to invoke elevated temperature and *p*CO_2_ as the primary drivers of elevated herbivory at our PETM site, our data do not support this. Reconstructed mean annual temperature (MAT) and leaf area damaged are not significantly correlated (Fig. 7; linear regression *R* ^2^ = 0.18, *P* = 0.34, *n* = 7 for the raw data; linear regression *R* ^2^ = 0.23, *P* = 0.34, *n* = 6 for the resampled data), although sample size is low. Precise estimates of paleo‐ *p*CO_2_ are currently impossible, but we would expect *p*CO_2_ to be strongly correlated with MAT, given the timescales considered (fossil leaf compression assemblages probably represent hundreds to thousands of years, whereas the residence time of CO_2_ in the atmosphere is <4 years). Furthermore, if temperature and *p*CO_2_ were dominant factors driving leaf area damaged, one would expect herbivory at Fifteenmile Creek (EECO site, MAT = 22.2 ± 2°C) to be similar to Hubble Bubble (PETM site, MAT = 20.1 ± 2.8°C), rather than comparable to the other sites.

The PETM represents an interval of geologically rapid environmental change in comparison with the other sites, but it is also unlikely that the quicker rate of environmental change is responsible for the elevated herbivory at Hubble Bubble. The Hubble Bubble flora lived ~100 ky after the major increase in *p*CO_2_ that occurred over a few millennia at the onset of the PETM. Although the *p*CO_2_ increase at the PETM onset was probably much faster than any known to occur during the EECO, PETM carbon cycle and climate change were still slow compared with ecological processes and the generation times of plants and insects. Studies of the most recent glacial–interglacial transition (21,000 years ago to present) demonstrate that plant and insect species underwent drastic changes in their geographic ranges in response to warming and ice sheet retreat (Coope 1995; Williams et al. 2004). Significant correlations exist between climate and vegetation records during this time interval, with typical vegetation lag times under 100 years (Williams et al. 2002). Therefore, it is reasonable to expect that plant and insect migrations were as capable of keeping pace with PETM climate change as they would have been with that during EECO.

Differences in other climate parameters between the PETM and the EECO have not yet been documented. Paleobotanical estimates of mean annual precipitation in the Bighorn Basin are similar during the PETM and EECO (Wing et al. 2006; Peppe et al. 2011; Diefendorf et al. 2015), and so variation in herbivory is not likely to be explained by differences in drought regime, which can affect leaf sugar and nitrogen levels (Mattson and Haack 1987; Koricheva et al. 1998). It is currently unknown, though, whether there were differences in seasonality between the PETM and EECO, and this may be an important factor affecting plant–herbivore interactions.

The most profound difference observed between Hubble Bubble and all other sites included in this analysis is the prevalence of plant species that have symbiotic associations with nitrogen‐fixing bacteria (Table 5), represented in our floras by legumes and *Alnus* (Quispel 1954). Chi‐squared tests indicate that the proportion of leaves from nitrogen‐fixing species (*χ* ^2^ = 554.3, df = 5, *P *<* *0.01) and the area of leaf tissue belonging to nitrogen‐fixing species (*χ* ^2^ = 2735.9, df = 3, *P *<* *0.01) both vary significantly among sites. Only Hubble Bubble has more leaves of nitrogen fixers than would be expected under the null hypothesis (665 observed vs. 373.5 expected counts). Looking across the six sites for which we have data, there is a significant, positive correlation between the proportion of leaves belonging to nitrogen‐fixing species and resampled leaf area damaged (Fig. 7B; linear regression *R* ^2^ = 0.81, *P* = 0.01, *n* = 6).

**Table 5:** Nitrogen‐fixing species

Flora	% leaves of N_2_‐fixers	% leaf area of N_2_‐fixers	% area leaf damaged, N_2_‐fixers	% leaf area damaged, non‐N_2_‐fixers	Ratio of % leaf area damaged on N_2_‐fixers: non‐N_2_‐fixers
Bonanza	31.3	6.8	0.75	2.27	~1:3
Republic	47.4	30.8	2.38	3.01	~4:5
Fifteenmile Creek	38.0	37.0	3.5	2.7	~4:3
PN	30.7	23.3	2.1	2.2	~1:1
Hubble Bubble	65.8	54.1	1.4	5.6	~1:4
Daiye Spa	16.6	13.9	6.5	3.3	~2:1

Our results suggest that nitrogen availability, or perhaps nutrient availability in general, regulates the opposing effects of elevated temperature and *p*CO_2_ on insect abundance and thereby total insect consumption (Fig. 1). Plant nitrogen content is the most important determinant of insect herbivore larval performance (Mattson 1980; Scriber and Slansky 1981), and we therefore hypothesize that the abundance of legumes during the PETM decreased nitrogen limitation across the ecosystem and buffered herbivores from *p*CO_2_‐induced decreases in leaf nutritional quality. Legume remains incorporated into the PETM soils would have provided a source of accessible nitrogen for all plants, facilitating higher leaf nitrogen concentrations across species. This would enable generalist herbivores to meet their nitrogen requirements and either maintain or increase insect population numbers. Thus, increased herbivory during the PETM is more likely attributable to higher insect abundances rather than to compensatory feeding.

A comparison of herbivory on N_2_‐fixing plants versus non‐N_2_‐fixing plants at each site supports our hypothesis (Table 5). While sample size is small, our data suggest an inverse relationship between prevalence of N_2_‐fixers in an ecosystem and preferential feeding on these plants (Fig. 7C). As the proportion of N_2_‐fixing leaves increases, the ratio of herbivory on N_2_‐fixers versus non‐N_2_‐fixers decreases. This is particularly apparent at Hubble Bubble, where N_2_‐fixing plants had only one‐fourth as much of their leaf area damaged as did non‐N_2_‐fixers. The relatively high concentration of biologically accessible nitrogen in that ecosystem would have decreased insects' dependence on legumes to fulfill their nutritional requirements. In contrast, Daiye Spa, which has the lowest abundance of nitrogen‐fixing plants, has twice as much herbivory on its legume species as on non‐N_2_‐fixing species.

The abundance of legumes in the PETM supports the widespread hypothesis that legumes' ability to form symbiotic relationships with N_2_‐fixing bacteria creates a competitive advantage over non‐N_2_‐fixing C3 plants in a high *p*CO_2_ world (e.g., Rogers et al. 2009). Increases in final dry mass under elevated *p*CO_2_ have been observed in woody legume saplings (Cernusak et al. 2011) and soybeans (Morgan et al. 2005; Rogers et al. 2006). In a study of annual herbaceous plants grown at elevated *p*CO_2_, Miyagi et al. (2007) observed greater enhancement in seed production in legumes than in non‐N_2_‐fixers and concluded that seed production is strongly limited by nitrogen supply. Few studies have been conducted that document the responses of field‐grown legumes to elevated *p*CO_2_ (Rogers et al. 2009), and those that have suggest that legumes in natural ecosystems are less responsive than those in managed systems to the abundance of carbon (van Groenigen et al. 2006). Our 56‐million year‐old natural laboratory provides one example of legume dominance in a high‐temperature, high‐*p*CO_2_ world. The fact that legumes are not dominant during the similarly warm and CO_2_‐rich EECO suggests the importance of site‐specific factors, including soil moisture and nutrient availability, on plant community composition. Unfortunately, it is currently impossible to constrain these in the fossil record.

### Insect herbivory through time

Leaf area damage at Hubble Bubble is higher than any value previously reported in the fossil record (Currano 2013), but it is lower than commonly cited estimates for modern forests. Coley and Aide (1991) reported average percent damage per year of 10.9% for tropical forests and 7.5% for temperate forests, and Coley and Barone (1996) obtained 14.2% for tropical dry forests and 7.1% for temperate broad‐leaved forests. A variety of factors may be responsible for the difference between these values and our Paleogene ones. First, herbivory may truly increase through time, due to insect diversification (Nicholson et al. 2015). The deeper time fossil record contradicts this, however. Percent leaf area damaged on fossil floras from the Early Permian (299–272 Ma) of Texas and Brazil fall within the range of our non‐PETM sites (Beck and Labandeira 1998; Adami‐Rodrigues et al. 2004; Labandeira and Allen 2007). Alternatively, a variety of factors specific to the fossil record may be responsible for underestimating Paleogene herbivory, including lower preservation chances for damaged leaves, the inability to sample completely consumed leaves, low preservation potential of very small damage types, and an over‐representation of canopy leaves, which often are tougher and have lower levels of herbivory than understory leaves (Lowman and Heatwole 1992; Barone 2000). We call for new taphonomic studies to quantify these possible biases. Last, insect herbivory today may truly be similar to that during the PETM, but the studies included in Coley and Aide's (1991) or Coley and Barone's (1996) literature reviews used methods that are not comparable to those used in fossil studies. This is supported by measurements of leaf area damaged on the uppermost layer of leaf litter, which provides a more accurate representation of what leaves become fossils than leaf samples measured while still on the tree. Adams et al. (2009) found 5.82% leaf area damaged at five lowland tropical forests and 5.48% at 86 temperate forest localities, which is only slightly higher than Hubble Bubble.

The PETM is the best geologic analog for the changes to Earth's atmosphere and climate system caused by fossil fuel burning, but it is a “best case” scenario because rates of anthropogenic carbon emissions are at least an order of magnitude greater than those of the PETM (Cui et al. 2011; Bowen et al. 2015). At the coarse temporal scale currently known, there is no clear paleontological evidence that plants and insects were unable to keep pace with PETM climate changes, but we advise caution when extrapolating to a future of more rapid change. If plant and insect species respond individualistically to climate perturbations, including timing and direction of changes in range and abundance, plant species may be exposed to and consumed by herbivores against which they have no adequate defenses. Likewise, insect species may encounter new food sources to which they are not as well adapted, resulting in increased feeding to fulfill nutritional requirements. The difference in herbivory at the PETM and EECO sites supports the varying results of laboratory and ecological studies and reinforces the importance of other factors, particularly nitrogen availability, in moderating insect folivory. Our paleontological work demonstrates the need for new ecological and agricultural studies on insect abundance, consumption, and fertilizer use as we prepare for a future of ever increasing *p*CO_2_ and temperature.

## Conflict of Interest

None declared.
